# Multi-frequency amplitude-programmable metasurface for multi-channel electromagnetic controls

**DOI:** 10.1515/nanoph-2022-0764

**Published:** 2023-03-01

**Authors:** Rui Yuan Wu, Shi He, Jun Wei Wu, Lei Bao, Tie Jun Cui

**Affiliations:** Institute of Electromagnetic Space, Southeast University, Nanjing 210096, China; State Key Laboratory of Millimeter Waves, Southeast University, Nanjing 210096, China; Key Laboratory of Optoelectronics Technology Ministry of Education, Beijing University of Technology, Beijing 100124, China; Pazhou Laboratory, Huangpu, Guangzhou 510555, China

**Keywords:** amplitude control, multi-frequency, programmable metasurface

## Abstract

The digital and programmable metasurfaces, as opposed to conventional metasurfaces, offer a more sophisticated method of collaborating information and physics, showcasing several real-time controls to electromagnetic (EM) ways in succinct ways. In this work, we propose a multi-frequency amplitude-programmable (MFAP) metasurface with multiple frequency channels to enhance the presentation and manipulation of EM data. With this metasurface, the reflected amplitudes can be simultaneously and independently encoded between high (digit “1”) and low (digit “0”) levels. The amplitude code is unique, which exhibits both reflection coefficients and radiation patterns to allow for flexible multi-functional EM operations with frequency. For instance, the MFAP metasurface can be used to design innovative communication systems by transmitting various EM signals individually across the channels in time domain. It is also possible to carry out multi-bit transmissions by mixing these frequency channels. By introducing complex coding patterns in space domain, it is possible to manipulate EM powers with greater precision. A square-split ring meta-atom that can achieve stable single-frequency amplitude control and multi-frequency 1 bit amplitude-programmable features is described as a proof-of-concept. Varactors loaded on metallic structures of various sizes are switched between operating states to modify the amplitude codes at each frequency channel. The suggested MFAP metasurface’s validity is confirmed by simulations and measurements from a dual-channel MFAP metasurface prototype.

## 1 Introduction

Due to the powerful capability to manipulate electromagnetic (EM), metasurfaces have seen significant progress in both engineering and physical science over the past ten years [1–6]. The metasurfaces are substantially thinner than the working wavelength and exhibit two-dimensional (2D) morphologies as opposed to bulky metamaterials. As a result, in addition to having good qualities such as lightweight, simple integration, and conformal, they also have versatile EM control capabilities. The generalized Snell’s law [7], which ignored the effective medium theory and incorporated the idea of phase abrupt transitions, is the current source of inspiration for the majority of metasurface designs. In recent years, it has been possible to arbitrarily customize EM waves by altering the size, shape, and orientation of the subwavelength meta-atoms by extending the metasurfaces to amplitude, polarization, and frequency abrupts [8–12]. Many EM manipulation features, such as beam forming, beam steering, EM holograms, polarization converter, and other techniques that are challenging to implement with natural materials, have been made possible by the introduction of metasurfaces.

Building a link between the EM physical and digital information [13–17], Cui et al. reported a ground-breaking concept of coding digital and programmable metasurfaces [18] in 2014. Two meta-atoms with a 180° phase difference are represented as the digital states “0” and “1” in physics in the most basic 1-bit phase coding scheme. The digital coding metasurface can alter the EM performance and gather the required information by arranging the 1 bit meta-atoms following the pre-designed coding sequences or patterns. By using a finer phase difference of 360°/2^*n*^, it is simple to apply this principle to the n-bit case, which enables convolution [19] and addition [20] operations on the coding patterns to implement more complex electromagnetic phenomena, such as multi-beam radiations and large-angle beam deflections with little distortion [21–23]. More importantly, this discretized approach makes it easier for programmable metasurfaces to transition from digital coding metasurfaces [18]. The bias voltages on loaded varactors or PIN diodes can be modulated to change the exposed digital state. To alter the coding patterns in real time, the field-programmable gate array (FPGA) is used to vary the EM manipulating function of the programmable metasurface. In low-cost antenna arrays, imaging, radar, and communication systems, this characteristic has received extensive researches [24–28]. Additionally, a new space-time-coding metasurface makes it possible to manipulate harmonic waves more flexibly, allowing for things like amplitude control and non-reciprocity reflection [29–32].

Studies of other EM properties, in addition to phase coding, are crucial for the development of programmable metasurfaces. The digital values “0” and “1” in the amplitude coding scheme always denote high or low scattered powers, respectively [33], which can be used directly to describe EM reflections and transmissions [34–37]. The amplitude coding scheme is simpler to produce and is more easily recognized by the receiver than the phase coding method. Additionally, multi-feature digital EM controls enable trickier operations in several dimensions [38–43]. Several amplitude-phase controllable and programmable metasurfaces that conduct power-adjustable and multi-beam radiations are suggested in Refs. [38, 39]. To establish dynamical polarization conversions, polarization-distinguished programmable metasurfaces are investigated in Ref. [40] for anisotropic properties. In multi-frequency amplitude-programmable metasurface, the design reported in Ref. [42] performs quite well. The metallic structures contain two PIN diodes, and the resonances (reflected amplitude digit “0”) at three frequencies are represented by the three working states “00,” “01/10,” and “11” respectively. The meta-atom in this design, however, can only display one digital state at a time. In other words, only by combining several units with various states are the controls of numerous frequencies completely independent at the unit level.

**Figure 1:**
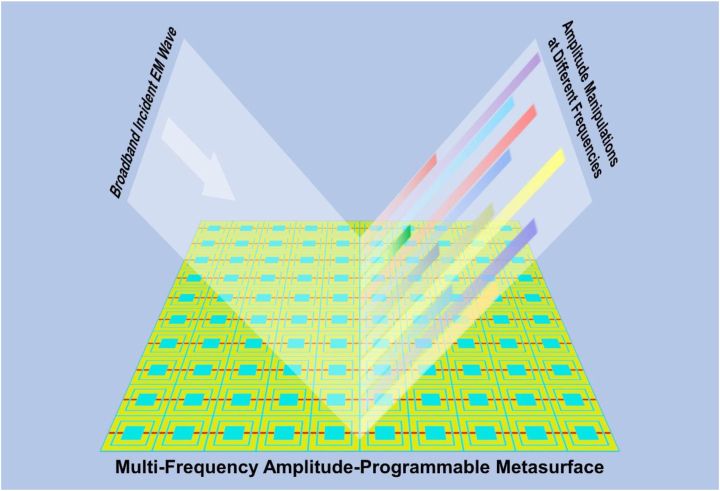
Schematic diagram of the EM manipulations at various frequencies via the MFAP metasurface.

To implement multi-channel EM manipulations, we propose a new multi-frequency amplitude-programmable (MFAP) metasurface in this study. According to Figure 1, by modulating the operating states of the varactors on the metallic square split rings in various diameters, the reflected amplitudes at various frequencies can be independently changed between the “0” and “1” states digitally in real time. As a result, several unusual features that are difficult to implement for metasurfaces operating at a single frequency can be accomplished, such as independent multi-channel EM power and information manipulations and the production of multi-bit digits in frequency domain. The potential uses in frequency-multiplexing communications and radar systems are shown by the results of the simulation, fabrication, and measurement of a dual-channel MFAP metasurface prototype.

## 2 Design of MFAP meta-atom

**Figure 2:**
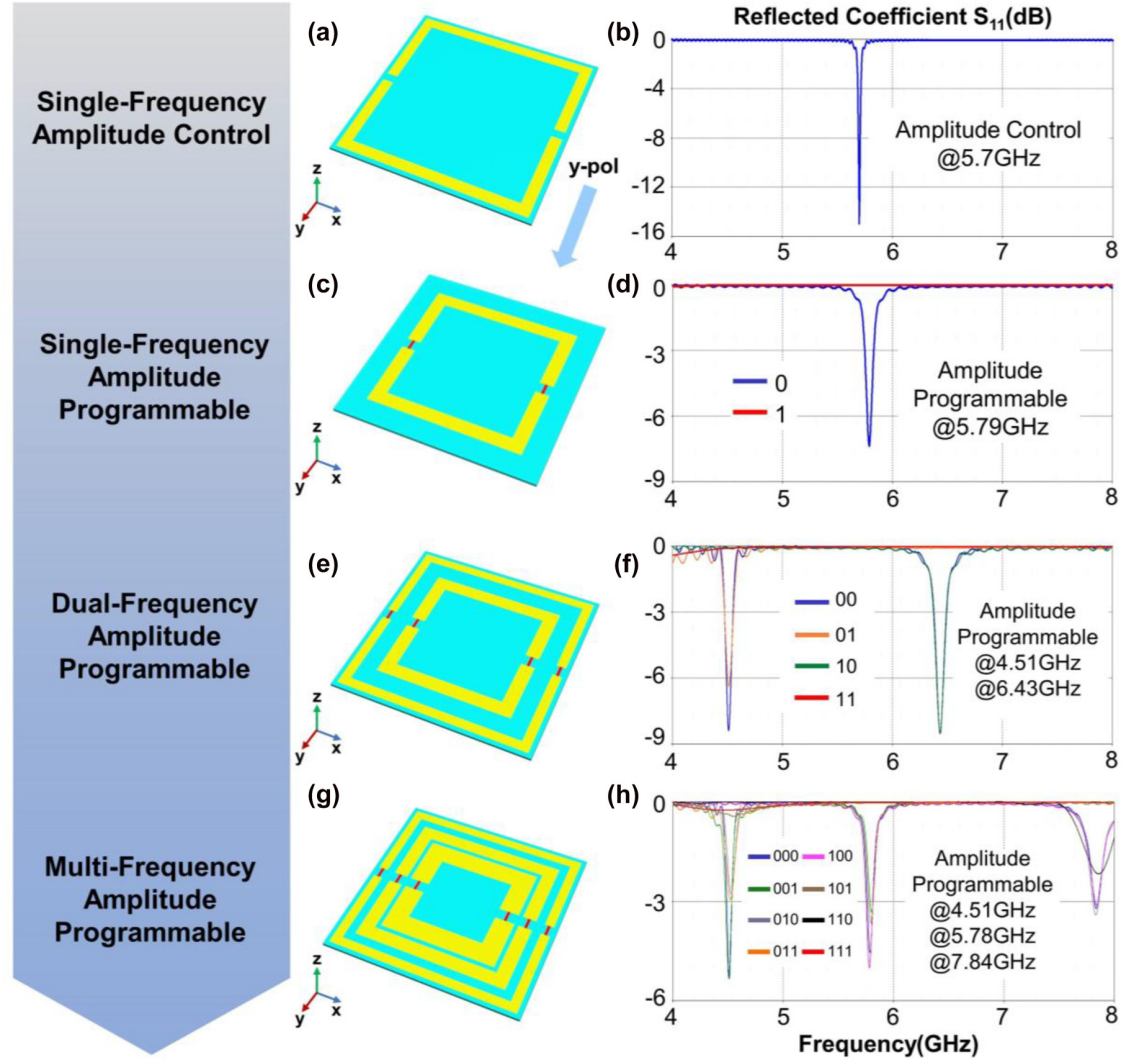
Evolution from stable single-frequency amplitude-controllable metasurface to MFAP metasurface. (a) and (b) The structure and reflected the performance of the single-frequency amplitude-controllable metasurface (ring size: 15 mm × 1.2 mm, split size: 1 mm); (c) and (h) the structures and reflected performances of the single-frequency (ring size: 17 mm × 1.5 mm), dual-frequency (ring sizes: 21 mm × 1 mm; 15 mm × 1.5 mm), and triple-frequency (ring sizes: 21 mm × 1 mm; 17 mm × 1.5 mm; 13 mm × 2.2 mm) amplitude-programmable metasurface.

The digital values “1” and “0” are used to encode the high and low levels of the reflected coefficient *S*_11_ in the amplitude-coding system. In order to make the receiver identify the signals from background noise, the reflected amplitude of digit “0” in the MFAP metasurfaces cannot be very close to zero. The multi-frequency property of the MFAP metasurface to achieve independent amplitude control at each frequency without mutual interference is the most important feature. To achieve this goal, our design is based on a straightforward meta-atom made of metallic square rings. As shown in Figure 2, all meta-atoms have a metallic ground plane with the size of 22 × 22 mm^2^, which are normally illumiated by a *y*-polarized plane wave. A special resonance occurs at 5.7 GHz to accomplish the amplitude control when two splits are carved in the center of a square ring, as shown in Figure 2a. Two varactors (designated SMV 2019-079LF in this study) are then incorporated into the splits, allowing the amplitude drop at 5.79 GHz to be dynamically regulated by altering bias voltages, as shown in Figure 2c and d. Since the size of the metallic structure in the meta-atom affects the resonant frequency, it is known that many square rings of various sizes (see captions in Figure 2) can be used to create multi-frequency features. Dual-channel (4.51 GHz, 6.43 GHz) and triple-channel (4.51 GHz, 5.78 GHz, and 7.84 GHz) MFAP performance can be obtained by arranging two and three rings, as shown in Figure 2e–h, after adjusting the widths and gaps of the rings. As a result, by applying the regulating signals, the amplitude responses at each frequency can be individually and dynamically determined.

**Figure 3:**
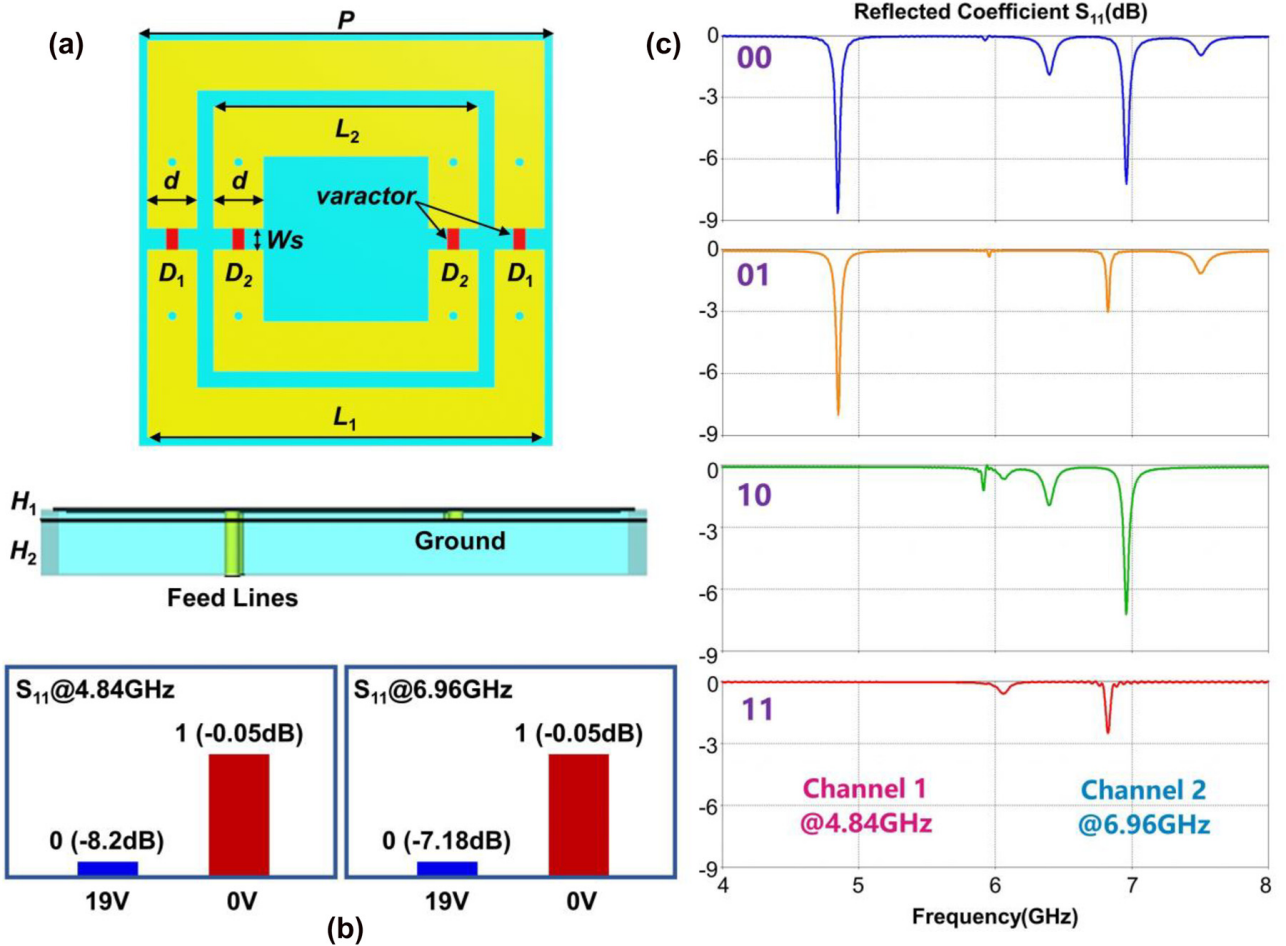
Structure and properties of the dual-channel MFAP meta-atom. (a) The front and side views; (b) and (c) the 1 bit amplitude programmable properties at dual frequencies 4.84 GHz and 6.96 GHz.

We take into account a practical dual-channel MFAP design to show the above concept’s strong ability while considering the complexity of fabrication. The improved meta-atom, which can operate 1 bit amplitude controls at 4.84 GHz and 6.96 GHz by modulating the voltages on the varactors in the outer and inner rings, is depicted in Figure 3a. The upper halves of the rings are connected with the ground plane through holes, as shown in the side view in Figure 3a. A feed-line layer is etched behind the ground plane and connected to the bottom halves of rings through holes to produce bias voltages. The substrate is made of F4B with the relative permittivity = 2.65 and thickness of *H*_1_ = 0.254 mm, which is only 0.004*λ* at 4.84 GHz and 0.006*λ* at 6.96 GHz. *H*_2_ is the thickness of the isolation substrate between the ground and feed lines, which can be arbitrarily chosen as per the design requirements. For example, we define *H*_2_ = 1.5 mm for convenience in fabrication. If a smaller thickness is required in some cases, it can also be 0.254 mm. Due to its thinness, the design can be applied to both integrated applications and some challenging circumstances. Other geometrical parameters include: the periodicity of meta-atom is *P* = 25 mm, the sizes of the outer and inner rings *L*_1_ and *L*_2_ are 21 mm and 16 mm, and the width *d* is 3 mm, and the width of the split is *W*_s_ = 1.3 mm considering the package of varactors. We select two kinds of bias voltages viz. 0 V and 19 V according to the equivalent series resonant circuit [44], in which the values of capacity, inductance, and resistance are 2.31 pF, 0.7 nH, 4.51 Ω and 0.22 pF, 0.7 nH, 2.38 Ω, respectively. Figure 3b illustrates the dual-channel 1 bit MFAP characteristics. When the voltage on *D*_1_ is *V*_D1_ = 19 V, there is a resonance with the amplitude drop of *S*_11_ = −8.2 dB at 4.84 GHz, which indicates the amplitude code “0”. When it is converted to 0 V, the resonance is disappeared with *S*_11_ = −0.05 dB, and the amplitude is encoded to “1.” Similarly, the reflected amplitude *S*_11_ at 6.96 GHz is −7.18 dB when *V*_D2_ = −19 V and −0.05 dB when *V*_D2_ = 0 V. Further, due to the independence between the manipulations at two frequencies, we performed 2 bit amplitude coding in frequency domain. As shown in Figure 3c, when the group of *V*_D1_ and *V*_D2_ is 19 V/19 V, 19 V/0 V, 0 V/19 V, and 0 V/0 V, we obtain four digital states of the 2-bit case “00,” “01,” “10,” and “11” by combining the two frequencies. To increase the information capacity, it can be further promoted to other states utilizing the tri-band metasurface in Figure 2h. Thus, frequency-multiplexing multi-bit EM controls are accomplished in the common meta-atom in addition to multi-channel EM manipulations being possible without mutual interference.

## 3 Design of MFAP metasurface and real-time multi-channel EM controls

**Figure 4:**
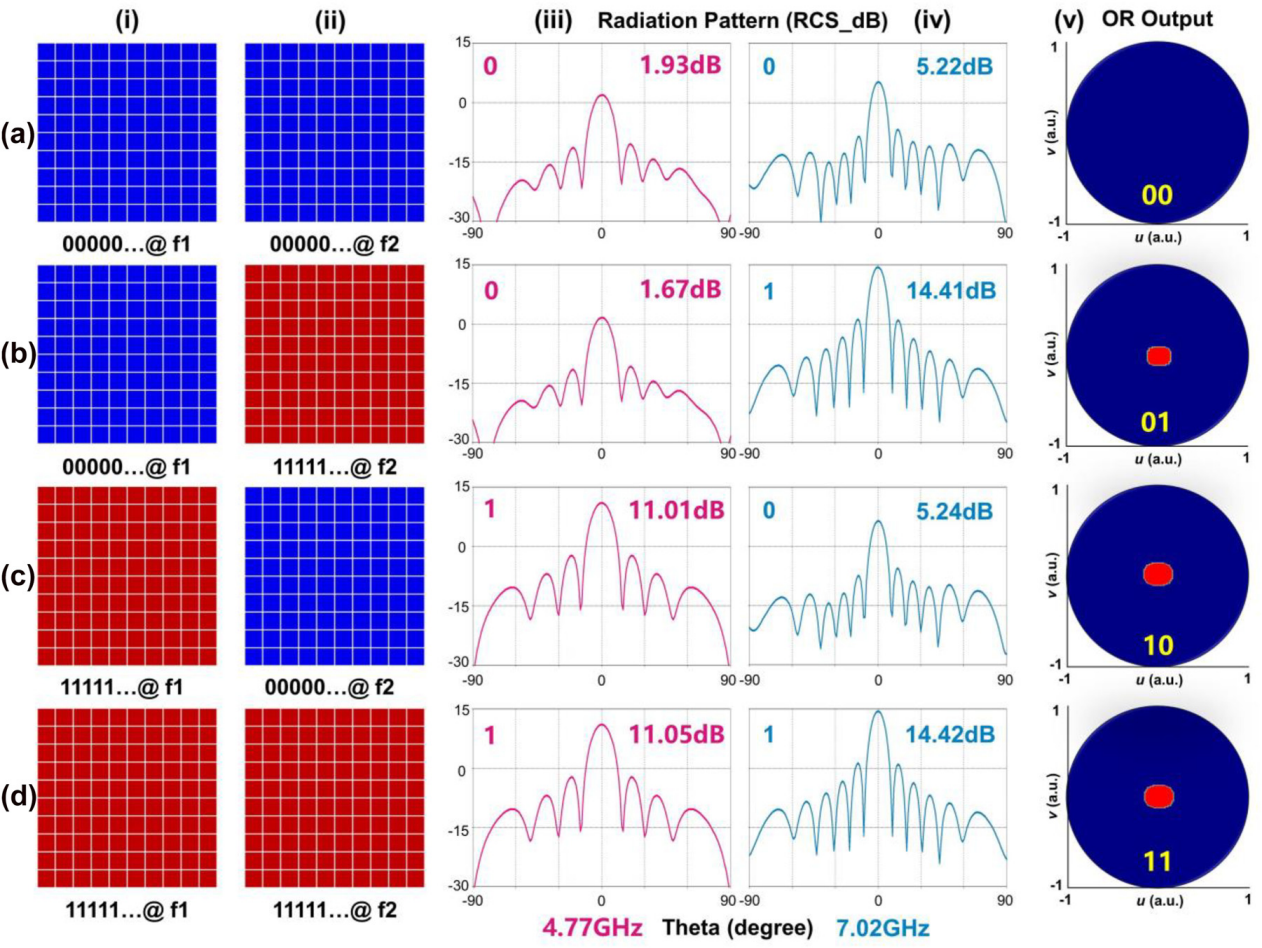
Scattering pattern controls of the MFAP metasurfaces. (a)–(d) The coding sequences and scattering patterns of the MFAP metasurfaces: (i) the coding sequences at channel *f*_1_; (ii) the coding sequences at channel *f*_2_; (iii) the scattering patterns at channel *f*_1_; (iv) the scattering patterns at channel *f*_2_; (v) the output of frequency-multiplexing OR logical operation in 2D radiation patterns in the *u*–*v* coordinate system (*u* = sin *θ* cos *φ*, *v* = sin *θ* sin *φ*).

One can take advantage of the MFAP features for more potent operations like multi-channel far-field EM information transmissions and power controls by arranging the meta-atoms in a metasurface aperture. For experiments, a dual-channel MFAP metasurface prototype with 10 × 10 meta-atoms is used (see Figure 1). Under the illumination of *y*-polarized plane waves in CST, the MFAP metasurface with different coding sequences “00000 … ” and “11111 … ” at two frequency channels (Figure 4i-ii) will generate the scattering patterns with different levels of Radar Cross Sections (RCSs). The variations in RCSs between the two coding sequences are roughly 9.3 dB at 4.77 GHz and 9.19 dB at 7.02 GHz, as shown in Figure 4a–d, demonstrating that the amplitude code can be readily distinguished from the scattering patterns. As a result, there are only minor variations in the operating frequency due to the coupling in array simulations, which allows the far-field results to remain consistent with the near-field results in Figure 3c.

It is important to note that this performance includes an EM domain inherent OR logical operation. Assuming an output indicator in the receiving part, as shown in Figure 4v, that can be lit (the output digit is “1”) under the condition that the coding sequences are “00000 … /11111 … ”, “11111 … /00000 … ”, and “11111 … /11111 … ” That is to say, at least one frequency channel has seen successful signal transmission. Due to the high level of isolation between the two channels, this phenomenon complies with the rules governing OR operations, which can increase the stability of the digital state “1”.

**Figure 5:**
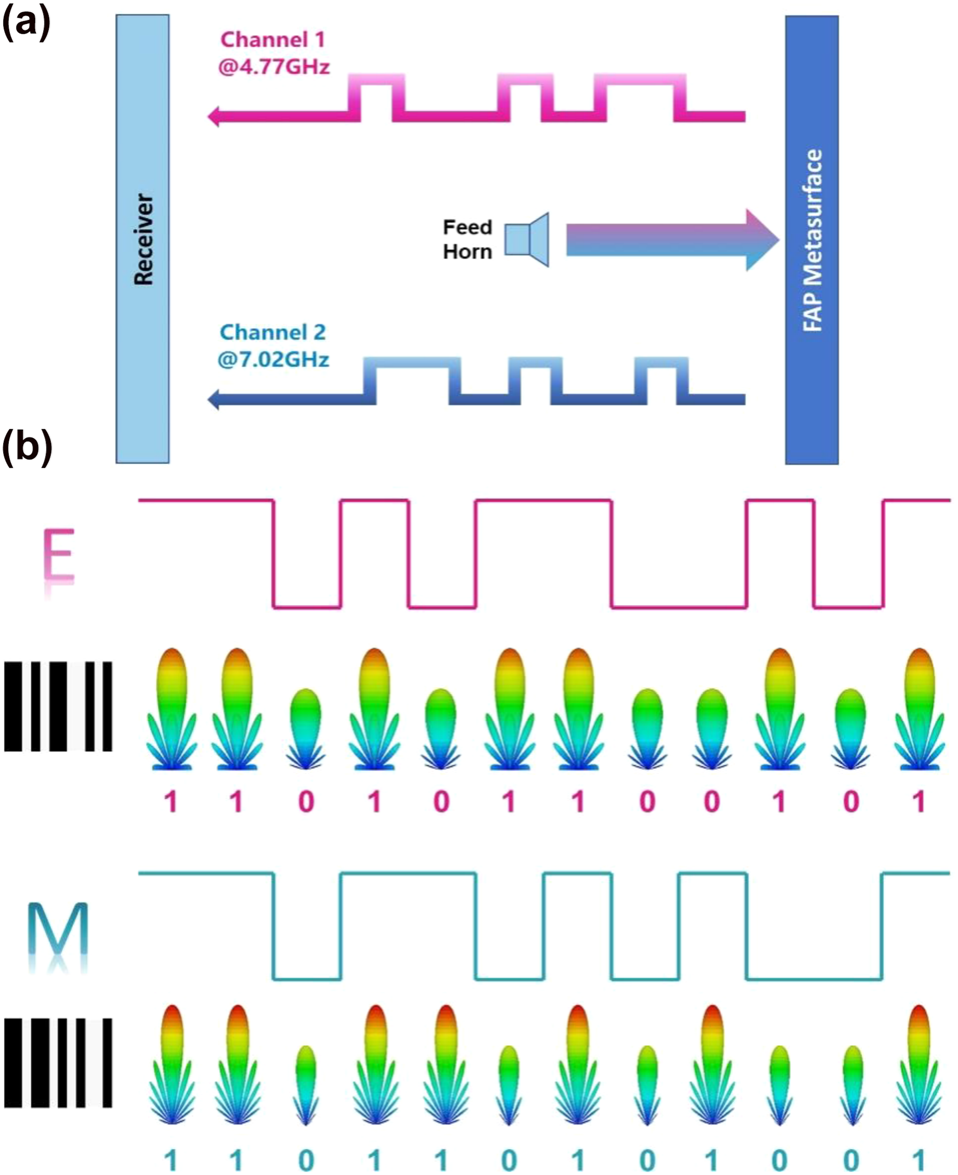
Schematic illustration of a dual-channel communication system via the MFAP metasurface. (a) Ideal model; (b) independent bar code transmissions of letter “E” and “M” at 4.77 GHz and 7.02 GHz in the far field.

We are motivated to create a novel architecture for a dual-channel communication system because when the EM signal broadcast in each frequency channel changes in time domain, the gain of the far-field pattern will change proportionally. The ideal model is depicted in Figure 5a, in which the feed horn’s broadband incidence can excite the data from the MFAP metasurface into free space. Without any interference, the two channels’ signals are sent simultaneously to the receiver. The variation tendency of the scattering patterns can also be used directly to get the conveyed information. As a result, it can be regarded as the spatial amplitude shift keying (ASK) approach with numerous benefits, including the straightforward design, high usage, straightforward demodulation, and so on. Additionally, its operation is almost similar to that of the direct information transmission system in Ref. [26], which avoids complex frequency mixing and modulation/demodulation and benefits from fewer receivers and a higher information capacity. Additionally, the process is also similar to how one-dimensional bar codes are made. Figure 5b shows how the letters “E” and “M,” whose coding sequences “110101100101” and “110110101001” can be broadcast directly in the two frequency channels, are represented by Code39 regulation. Future technology will allow for direct space transmission of complicated Quick Response (QR) Codes or the entire bar-code data set, including the start, check, and stop bits. We can further take all frequenciy channels into account for higher-bit information transmissions. For example, if we combine the dual channels together, the transmitted information is “11 11 00 11 01 10 11 00 01 10 00 11,” which is a 2-bit case. Similarly, 3-bit information transmissions can be conducted by combining three channels. This enables high-capacity and high-precision EM information transmissions. Because of the stable reflected phase responses, the receiver should be at the same level with the transmitter. By further introducing multi-band phase adjustment [43] and even amplitude-phase controllable scheme [12], more complicated systems for deflecting to multiple targets can be achieved.

**Figure 6:**
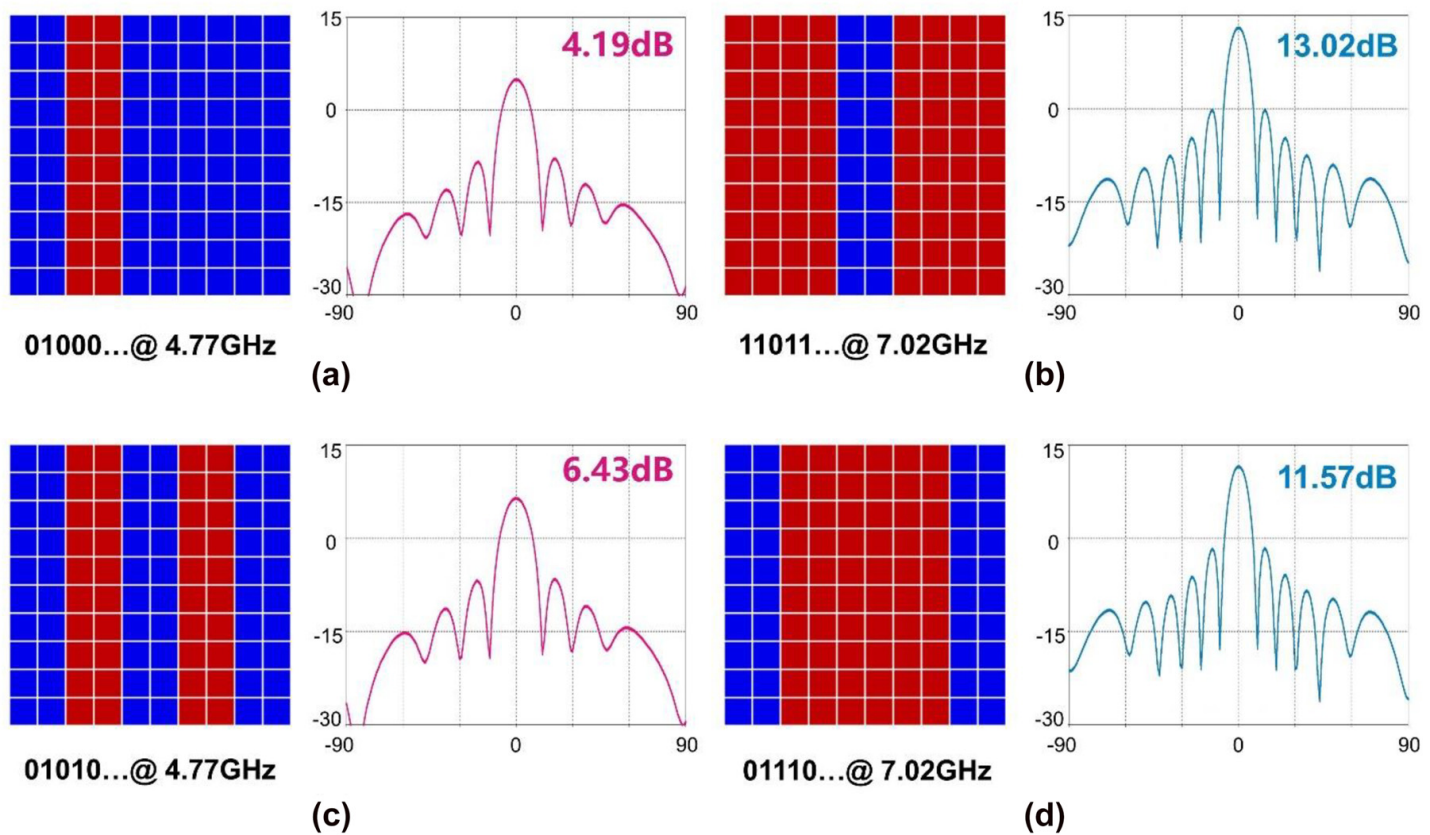
Power manipulations via the space-domain coding sequences. (a) “01000 … ” at 4.77 GHz; (b) “11011 … ” at 7.02 GHz; (c) “01010 … ” at 4.77 GHz; (d) “01110 … ” at 7.02 GHz.

As is well known, the radiating power is dependent on the metasurface reflection coefficient, specifically the proportion of “1” to “0”. Therefore, to obtain more accurate manipulations of the radiating powers, one can construct intricate coding sequences in the space domain on the metasurface aperture. The performance is comparable when all meta-atoms or columns are in control of the MFAP metasurface since the ratio of the two digital states is the crucial variable. Each frequency channel is designed with several common coding sequences, and the radiation patterns are depicted in Figure 6. This suggests a quick and effective way to change the output power. In detail, the coding sequences “01000 … ” and “01010 … ” are selected at 4.77 GHz in Figure 6a and c, whose proportion of “1” state on the aperture are 20% and 40%, corresponding to the powers of 4.19 dB and 6.43 dB, respectively. Another two coding sequences “11011 … ” and “01110 … ” are chosen at 7.02 GHz (Figure 6b–d), in which 80% and 60% meta-atoms on the aperture are of state “1,” resulting in the outputs of 13.02 dB and 11.57 dB. In accordance with the findings in Figure 4, one can state that by expanding the coding-pattern designs, the radiation powers can be independently modified from the least to the greatest levels.

**Figure 7:**
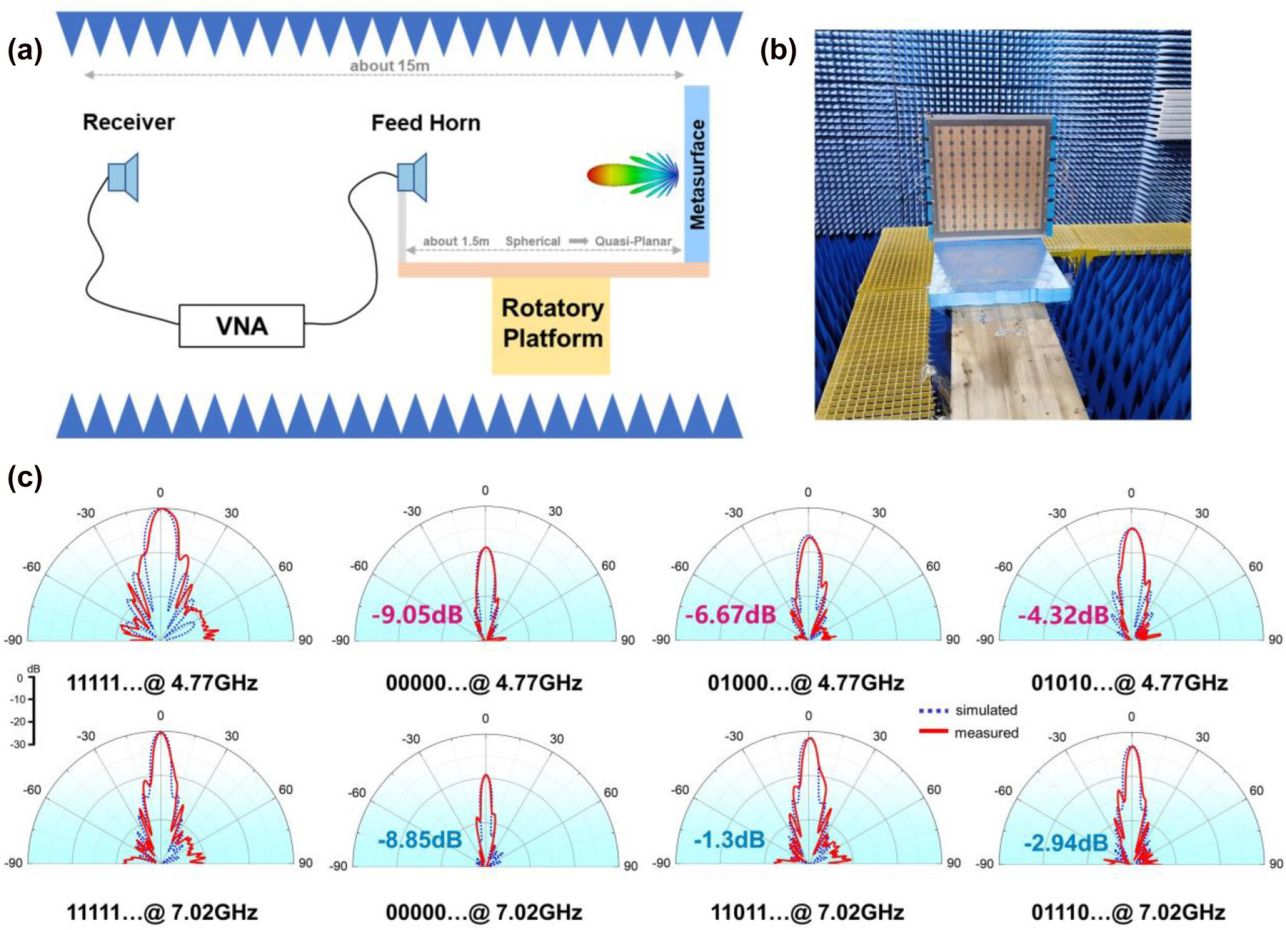
Experimental setup and measured results. (a) Schematic of the far-field experiments for the MFAP metasurface in the anechoic chamber. (b) The photos of the experimental setup and the fabricated MFAP metasurface prototype. (c) The simulated and measured radiation patterns of various coding sequences at 4.77 GHz and 7.02 GHz.

As shown in Figure 7b, we created and measured an MFAP metasurface sample to further confirm the aforementioned functions. The metasurface is regulated in a column due to the feed lines’ simplicity, as seen in Figure 6. The anechoic chamber is where the far-field experiments are carried out (Figure 7a). To provide a quasi-planar EM incidence, a C-band horn antenna serving as the feed source is positioned around 1.5 m away from the sample. They are both put on the rotating platform. The feed horn and Vector Network Analyzer (VNA) are both connected to the receiver, which is situated around 15 m away from the metasurface. The measured radiation patterns are shown in Figure 7c, and the coding sequences can be modified in real time. We use the gain of coding sequence “11111 … ” to normalize all results to show the power control effect. For the channel at 4.77 GHz, the power drops are 9.05 dB, 6.67 dB, and 4.32 dB when the coding sequences are “00000 … ”, “01000 … ” and “01010 … ”. Similarly, for the channel at 7.02 GHz, the power drops are 8.85 dB, 1.3 dB, and 2.94 dB when the coding sequences are “00000 … ”, “11011 … ” and “01110 … ”. Except for the beam widths being slightly wider and the side lobes being slightly larger than the simulated values due to the difference between the quasi and perfect plane-wave incidences, which has no impact on the functionality performance, Figure 7c, and Table 1 also demonstrate that the measured results closely match the simulations, which can prove the validity of the suggested MFAP metasurface.

**Table 1:** Comparison between simulated and measured results.

Frequency	Coding sequence	Simulatedpower drop	Measuredpower drop
4.77 GHz	11111 (normalized)	0 dB	0 dB
	00000	9.1 dB	9.05 dB
	01000	6.84 dB	6.67 dB
	01010	4.6 dB	4.32 dB
7.02 GHz	11111 (normalized)	0 dB	0 dB
	00000	9.19 dB	8.85 dB
	11011	1.4 dB	1.3 dB
	01110	2.85 dB	2.94 dB

## 4 Conclusions

To implement the multi-channel EM manipulations, we have demonstrated a unique MFAP metasurface. The reflected amplitude responses of the meta-atom at various frequencies can be adjusted between the 1-bit digital states “0” and “1” without interference by modulating the operating states of varactors on square-split rings in various diameters. More complex capabilities, such as EM power and information manipulations, have been carried out at the metasurface level based on this fundamental characteristic. A dual-channel MFAP metasurface is created to accomplish the 1-bit amplitude programmable capacity at 4.77 GHz and 7.02 GHz, which can be distinguished from near-field reflected coefficients and far-field radiations, to empirically test this theory. By adding extra functional channels, it can be further expanded to the frequency-multiplexing n-bit situation. The frequency domain replies also have a built-in OR logical operation that may identify whether or not a signal in two channels is present. We proposed a novel design for a dual-channel information transmission system, allowing for the simultaneous and independent transmission of two series of signals to the receivers. Furthermore, by endowing the coding sequences in the space domain, more accurate EM power controls can be obtained. The suggested MFAP metasurface’s viability can be confirmed by both simulated and measured outcomes, which paves the way for its further promotion to more channels for greater information capacity. Overall, by combining the thorough controls in the frequency, time, and space domains, our proposed MFAP can greatly enhance the EM manipulating functionalities. Future implementations of more complex system-level designs will offer excellent chances for frequency-multiplexing, multi-functional wireless communications, and radar applications.
